# Supplementary material for: Epidemiological surveillance of waterborne diseases among displaced populations: A cross-sectional study
Source: PLOS Glob Public Health. 2025 Dec 1;5(12):e0005527. doi: 10.1371/journal.pgph.0005527 (PMC12668538; doi:10.1371/journal.pgph.0005527)
Supplement: Attachment — Submitted filename: Response to Reviewers R1.docx [file pgph.0005527.s002.docx]

October 16, 2025

PLOS Global Public Health

Ref: PGPH-D-25-01756

Resubmission of manuscript "Epidemiological Surveillance of Waterborne Diseases Among Displaced Populations: A Cross-Sectional Study”

Dear Prof. Jenna Scaramanga,

Staff Editor

PLOS Global Public Health

Thank you for the opportunity to revise our manuscript. We appreciate the careful review and constructive suggestions. We believe that the document is substantially improved after making the suggested edits. Following this letter are the reviewers' comments with our responses in red, including how and where the text was modified. The revision has been developed in consultation with all coauthors, and each author has approved the final form of this revision.

Sincerely,

Dr. Samer Abuzerr

**Response to Reviewer(s)' Comments:**

Journal Requirements:

1. Please include a complete copy of PLOS’ questionnaire on inclusivity in global research in your revised manuscript. Our policy for research in this area aims to improve transparency in the reporting of research performed outside of researchers’ own country or community. The policy applies to researchers who have travelled to a different country to conduct research, research with Indigenous populations or their lands, and research on cultural artefacts. The questionnaire can also be requested at the journal’s discretion for any other submissions, even if these conditions are not met.  Please find more information on the policy and a link to download a blank copy of the questionnaire here: https://journals.plos.org/globalpublichealth/s/best-practices-in-research-reporting. Please upload a completed version of your questionnaire as Supporting Information when you resubmit your manuscript.

Done

2. In the online submission form, you indicated that Datasets are available from the corresponding author upon reasonable request, in accordance with data transparency and ethical guidelines.

All PLOS journals now require all data underlying the findings described in their manuscript to be freely available to other researchers, either 
1. In a public repository, 
2. Within the manuscript itself, or 
3. Uploaded as supplementary information.

This policy applies to all data except where public deposition would breach compliance with the protocol approved by your research ethics board. If your data cannot be made publicly available for ethical or legal reasons (e.g., public availability would compromise patient privacy), please explain your reasons by return email and your exemption request will be escalated to the editor for approval. Your exemption request will be handled independently and will not hold up the peer review process, but will need to be resolved should your manuscript be accepted for publication. One of the Editorial team will then be in touch if there are any issues.

Done

3. Please amend your detailed Financial Disclosure statement. This is published with the article. It must therefore be completed in full sentences and contain the exact wording you wish to be published.

If you did not receive any funding for this study, please simply state: “The authors received no specific funding for this work.

Done

4. Please send a completed 'Competing Interests' statement, including any COIs declared by your co-authors. If you have no competing interests to declare, please state "The authors have declared that no competing interests exist". Otherwise please declare all competing interests beginning with the statement "I have read the journal's policy and the authors of this manuscript have the following competing interests:"

Done

5. Please provide separate figure files in .tif or .eps format.

For more information about figure files please see our guidelines: 
https://journals.plos.org/globalpublichealth/s/figures 
<https://journals.plos.org/globalpublichealth/s/figures#loc-file-requirements>

Done

6. Some material included in your submission may be copyrighted. According to PLOS’s copyright policy, authors who use figures or other material (e.g., graphics, clipart, maps) from another author or copyright holder must demonstrate or obtain permission to publish this material under the Creative Commons Attribution 4.0 International (CC BY 4.0) License used by PLOS journals. Please closely review the details of PLOS’s copyright requirements here: PLOS Licenses and Copyright. If you need to request permissions from a copyright holder, you may use PLOS's Copyright Content Permission form.

Done

Please respond directly to this email or email the journal office and provide any known details concerning your material's license terms and permissions required for reuse, even if you have not yet obtained copyright permissions or are unsure of your material's copyright compatibility.

Potential Copyright Issues:
Figure 1: please (a) provide a direct link to the base layer of the map (i.e., the country or region border shape) and ensure this is also included in the figure legend; and (b) provide a link to the terms of use / license information for the base layer image or shapefile. We cannot publish proprietary or copyrighted maps (e.g. Google Maps, Mapquest) and the terms of use for your map base layer must be compatible with our CC-BY 4.0 license.

Note: if you created the map in a software program like R or ArcGIS, please locate and indicate the source of the basemap shapefile onto which data has been plotted.

If your map was obtained from a copyrighted source please amend the figure so that the base map used is from an openly available source. Alternatively, please provide explicit written permission from the copyright holder granting you the right to publish the material under our CC-BY 4.0 license.

Please note that the following CC BY licenses are compatible with PLOS license: CC BY 4.0, CC BY 2.0 and CC BY 3.0, meanwhile such licenses as CC BY-ND 3.0 and others are not compatible due to additional restrictions.

If you are unsure whether you can use a map or not, please do reach out and we will be able to help you. The following websites are good examples of where you can source open access or public domain maps: 
* U.S. Geological Survey (USGS) - All maps are in the public domain. (http://www.usgs.gov) 
* PlaniGlobe - All maps are published under a Creative Commons license so please cite “PlaniGlobe, http://www.planiglobe.com, CC BY 2.0” in the image credit after the caption. (http://www.planiglobe.com/?lang=enl) 
* Natural Earth - All maps are public domain. (<http://www.naturalearthdata.com/about/terms-of-use/>)

Thank you for your valuable comment. We have revised Figure 1 to ensure full compliance with the CC-BY 4.0 license requirements. The base layer of the Gaza Strip and its administrative boundaries was redrawn using openly available shapefiles from Natural Earth (public domain). The updated figure caption now includes a direct link to the data source and license information. The figure was created entirely by the authors using QGIS.

7. If the reviewer comments include a recommendation to cite specific previously published works, please review and evaluate these publications to determine whether they are relevant and should be cited. There is no requirement to cite these works unless the editor has indicated otherwise. 
Okay

[Note: HTML markup is below. Please do not edit.]

**Reviewers' comments:**

Reviewer's Responses to Questions

**Comments to the Author**

1. Does this manuscript meet PLOS Global Public Health’s [publication criteria](https://track.editorialmanager.com/CL0/https:%2F%2Fjournals.plos.org%2Fglobalpublichealth%2Fs%2Fcriteria-for-publication/1/010f019950d9d75b-ea80fa40-1699-4b0c-b132-6fcc77d6929b-000000/mRCvmAaUZ742caLqocn_c36AhngVJEQ8d3RfBxbsTN0=228)? Is the manuscript technically sound, and do the data support the conclusions? The manuscript must describe methodologically and ethically rigorous research with conclusions that are appropriately drawn based on the data presented.

Reviewer #1: Yes

Reviewer #2: Yes

2. Has the statistical analysis been performed appropriately and rigorously?

Reviewer #1: Yes

Reviewer #2: Yes

3. Have the authors made all data underlying the findings in their manuscript fully available (please refer to the Data Availability Statement at the start of the manuscript PDF file)?

The [PLOS Data policy](https://track.editorialmanager.com/CL0/https:%2F%2Fjournals.plos.org%2Fglobalpublichealth%2Fs%2Fdata-availability/1/010f019950d9d75b-ea80fa40-1699-4b0c-b132-6fcc77d6929b-000000/T8seIGVGN8Jj0aVZeTAuBe-4amX6unu0dgU5hsaU3-w=228) requires authors to make all data underlying the findings described in their manuscript fully available without restriction, with rare exception. The data should be provided as part of the manuscript or its supporting information, or deposited to a public repository. For example, in addition to summary statistics, the data points behind means, medians and variance measures should be available. If there are restrictions on publicly sharing data—e.g. participant privacy or use of data from a third party—those must be specified.

Reviewer #1: No

Reviewer #2: Yes

4. Is the manuscript presented in an intelligible fashion and written in standard English?

PLOS Global Public Health does not copyedit accepted manuscripts, so the language in submitted articles must be clear, correct, and unambiguous. Any typographical or grammatical errors should be corrected at revision, so please note any specific errors here.

Reviewer #1: Yes

Reviewer #2: Yes

**5. Review Comments to the Author**

Please use the space provided to explain your answers to the questions above. You may also include additional comments for the author, including concerns about dual publication, research ethics, or publication ethics. (Please upload your review as an attachment if it exceeds 20,000 characters)

Reviewer #1: I read the manuscript, "Epidemiological Surveillance of Waterborne Diseases Among Displaced Populations: A Cross-Sectional Study," which provides information on the prevalence of waterborne diseases in Gaza in the spring and summer months of 2025 among surveyed participants. The study also looks at the associated risk factors of these illnesses by using logistic regression and confirms the presence of contaminated water sources through water sampling at from displaced households. This study provides important information on the prevalence of waterborne illnesses in Gaza among the displaced populations. I believe much of the study is conducted well, but there are a few areas for potential improvement. First, I believe the authors should more explicitly define the candidate variables included for the bivariate and logistic regression analyses, including justification of the selected parameters. Also, a detailed map showing the prevalence of the surveyed illnesses would be very interesting to see. Similarly, mapping the reliance on different water sources, latrine access, etc. would add some insights. However, I understand the sensitivity of such information and concede making the data public may be an issue. Additionally, it may be good to include the possibility of interventions for waterborne illness being made on the basis of predictive intelligence employing satellite remote sensing of environmental conditions associated with waterborne diseases, like cholera (https://doi.org/10.1038/s41598-022-22946-y). The authors mention the possibility of targeted interventions relying on real-time data, and this approach could supplement their efforts in areas of high displacement, such as Gaza, where data is limited. On a minor note, double check grammar errors in the manuscript. One that I noted was at the conclusion start "In concludsion."

Authors should more explicitly define the candidate variables included for the bivariate and logistic regression analyses, including justification of the selected parameters.

Thank you for this valuable suggestion. We have revised the **Methods (Statistical Analysis)** section to provide a clearer description of the candidate variables included in the bivariate and multivariable logistic regression models, along with justification for their inclusion. Specifically, we now indicate that variables were selected based on epidemiological relevance and findings from prior literature on waterborne disease risk in displaced or humanitarian settings. These include age, sex, water source type, sanitation access, soap availability, and household crowding.

A detailed map showing the prevalence of the surveyed illnesses would be very interesting to see. Similarly, mapping the reliance on different water sources, latrine access, etc. would add insights.

We thank the reviewer for this valuable suggestion. Due to data sensitivity and security considerations in Gaza, we are unable to display precise spatial locations of shelters or households. However, in response to the reviewer’s recommendation, we have added a new Supplementary Table 3 that presents governorate-level aggregated data on the prevalence of reported waterborne illness symptoms, primary water sources, and sanitation conditions.

This table provides a clear overview of regional variations while maintaining participant confidentiality and ensuring compliance with ethical data protection standards. The findings highlight significant disparities across governorates, with higher prevalence of waterborne symptoms and reliance on unregulated water sources observed in North Gaza and Deir al-Balah.

It may be good to include the possibility of interventions for waterborne illness being made on the basis of predictive intelligence employing satellite remote sensing of environmental conditions associated with waterborne diseases, like cholera

We agree that predictive intelligence and satellite-based surveillance represent promising tools for early detection and intervention. We have now incorporated a discussion of this approach in the Discussion section, citing the suggested reference and linking it to our findings on the need for real-time surveillance.

Minor grammatical errors throughout the manuscript (e.g., ‘In concludsion’).

Thank you for noting this. The entire manuscript has been carefully proofread, and all typographical and grammatical errors have been corrected (including “In concludsion,” which now reads “In conclusion”).

Reviewer #2: In this study, the authors aim to investigate how conflict-driven displacement in Gaza affects access to safe water, sanitation, and hygiene (WASH) and how these factors contribute to the burden of waterborne diseases. By combining survey data with microbiological testing of household water samples, the authors demonstrate the link between unsafe water, inadequate sanitation, and poor hygiene with increased risk of disease. In this study authors tried to generate evidence that underscores the urgent need for integrated WASH interventions and improved disease surveillance to protect vulnerable displaced populations in conflict-affected settings.

However, the article has some limitations, which are mentioned below:
1) The selection process for participants needs to be described in greater detail.

We appreciate this important comment. The participant selection process has now been described in more detail in the Methods – Study Design and Sampling section. Specifically, we clarified how households and individuals were selected using a stratified cluster sampling approach within displacement shelters.

2) Additional references are required to support key statements. For example, the claim “While numerous studies have documented the heightened risk of waterborne diseases in displacement settings globally, including in regions such as Yemen, South Sudan, and Syria, limited data exist from the Gaza Strip under conditions of ongoing siege and acute infrastructural collapse” should be backed with citations. Several other statements throughout the manuscript also lack appropriate references.

We agree and have now added appropriate peer-reviewed references to strengthen this statement and other similar ones throughout the Introduction.

3) In the Methods section, the authors state that displacement shelters were selected based on population density and accessibility. However, no details—such as threshold values or specific methods used—are provided for this selection process.

We have now clarified this aspect in the Methods – Sampling Procedure section.
4) The terms individuals and households appear to be used interchangeably without clarification. It should be specified whether only one individual was selected per household, and this detail should be explicitly cited in the text.

We thank the reviewer for noting this inconsistency. We have revised the text throughout the Methods and Results sections to maintain consistency. A clarification was added that one adult respondent represented each household, reporting on the household’s WASH conditions and all members’ symptoms.
5) The line “Variables with a p-value < 0.2 in bivariate analysis were included in the multivariate model” is redundant, as it was already mentioned in the previous paragraph.

We agree. The redundant sentence was deleted from the Data Analysis subsection to streamline the description of the statistical methods.
6) The study notes that the presence of children under five years showed “borderline significance.” This should be clarified, as statistically it appears to be non-significant.

We appreciate this clarification. We have revised the Results section to explicitly state that the association was not statistically significant (p = 0.08) and adjusted the language accordingly.

6. PLOS authors have the option to publish the peer review history of their article ([what does this mean?](https://track.editorialmanager.com/CL0/https:%2F%2Fjournals.plos.org%2Fglobalpublichealth%2Fs%2Feditorial-and-peer-review-process%23loc-peer-review-history/1/010f019950d9d75b-ea80fa40-1699-4b0c-b132-6fcc77d6929b-000000/cW5lrGNRGa_3kshrLzSiJVe6pe7PLiOB8uHxrHTLEYU=228)). If published, this will include your full peer review and any attached files.


**Do you want your identity to be public for this peer review?** If you choose “no”, your identity will remain anonymous but your review may still be made public.


For information about this choice, including consent withdrawal, please see our [Privacy Policy](https://track.editorialmanager.com/CL0/https:%2F%2Fwww.plos.org%2Fprivacy-policy/1/010f019950d9d75b-ea80fa40-1699-4b0c-b132-6fcc77d6929b-000000/AHuANKWxJcIHhJnumXQ4DV1LzFRCXvrktsZYOhcoCPI=228).

Reviewer #1: No

Reviewer #2: No

[NOTE: If reviewer comments were submitted as an attachment file, they will be attached to this email and accessible via the submission site. Please log into your account, locate the manuscript record, and check for the action link "View Attachments". If this link does not appear, there are no attachment files.]

While revising your submission, please upload your figure files to the Preflight Analysis and Conversion Engine (PACE) digital diagnostic tool, https://pacev2.apexcovantage.com/. PACE helps ensure that figures meet PLOS requirements. To use PACE, you must first register as a user. Registration is free. Then, login and navigate to the UPLOAD tab, where you will find detailed instructions on how to use the tool. If you encounter any issues or have any questions when using PACE, please email PLOS at figures@plos.org. Please note that Supporting Information files do not need this step.
